# Spirulina Enhances Bone Modeling in Growing Male Rats by Regulating Growth-Related Hormones

**DOI:** 10.3390/nu12041187

**Published:** 2020-04-24

**Authors:** Jin Ah Cho, Seong Yeon Baek, Sun Hee Cheong, Mee Ree Kim

**Affiliations:** 1 Department of Food and Nutrition, Chungnam National University, 99, Daehak-ro, Yuseong-gu, Daejeon 34134, Korea; jacho@cnu.ac.kr (J.A.C.); qor7683@naver.com (S.Y.B.); 2 Department of Marine Bio Food Science, College of Fisheries and Ocean Science, Chonnam National University, Yeosu 550-749, Korea; sunny3843@jnu.ac.kr

**Keywords:** spirulina, bone modeling, bone strength, parathyroid hormone, growth hormone, antioxidant

## Abstract

In recent years, growth hormone deficiency in children has been treated with hormone therapy despite the possible significant side effects. Therefore, it was deemed beneficial to develop functional foods or dietary supplements for safely improving children’s growth. *Spirulina platensis* is known for its high antioxidant, anti-aging, anti-cancer, and immunity-enhancing properties, as well as its high digestibility and high protein content, but little has been reported about its influence on bone development in children with a normal supply of protein. In this study, we evaluated the effects of spirulina on the bone metabolism and antioxidant profiles of three-week-old growing male rats. The animals were divided into four groups (*n* = 17 per group) and were fed AIN93G diets with 0% (control), 30% (SP30), 50% (SP50), and 70% (SP70) of casein protein replaced by spirulina, respectively, for seven weeks. We observed that spirulina enhanced bone growth and bone strength by stimulating parathyroid hormone and growth hormone activities, as well its increased antioxidant activity. These results indicate that spirulina provides a suitable dietary supplement and alternative protein source with antioxidant benefits for growth improvement in early developmental stages.

## 1. Introduction

Bone modeling is the process by which bone is either formed on an existing bone surface by osteoblasts without prior resorption or removed by osteoclasts (resorption modeling), and is the dominant process during skeletal growth [1]. Bone modeling is thus essential for the proper longitudinal growth, with cells in epiphyseal growth plates responsible for continued elongation of bones until the body’s full size is reached [2,3,4]. Therefore the epiphyseal growth plate of the iliac bone is the most useful indicator of bone growth, which is regulated by the activities of the growth hormone (GH) secreted by the anterior pituitary gland of the brain [5,6].

Insulin-like growth factor 1 (IGF-1) is a single-chain, 70 amino acid polypeptide that is mainly secreted by the liver and acts in an insulin-like manner. IGF-1 is also secreted by osteoblasts, and is considered an auto- or paracrine regulator of osteoblastic cell function [7]. GH, parathyroid hormone, and calcitriol also stimulate IGF-1 production during the growth phase [8]. Therefore, IGF-1 is recognized as a growth factor that has an important role in the maintenance of bone mass. This action eventually stimulates the synthesis of carbohydrates, lipids, and proteins in target tissues [9]. Although GH directly stimulates the proliferation and differentiation of osteoblast cells, IGF-1 also increases osteocalcin and collagen synthesis in osteoblasts, as well as differentiation of osteoblasts, thereby increasing bone formation and inhibiting collagenase expression.

Chronic protein deficiency has been reported to delay skeletal growth and bone maturity and cause fatty infiltration of the liver in infants and young children, conditions that are often associated with chronic malnutrition, a major factor affecting child morbidity and mortality in developing countries [10,11]. It has been reported that a low-protein diet reduces tibia length and diameter, and bone mineral content (BMC) in growing rats [12] and children [13]. Also low levels of circulating IGF-1 and the presence of a fatty liver are conditions frequently observed in children with a protein-deficient diet [14]. 

Spirulina (*Spirulina platensis*), a green spiral bacterium classified as a cyanobacterium, has been recognized by international organizations such as the WHO, FAO, and UNICEF as a dietary supplement (*FAO Fisheries and Aquaculture Circular*. No. 1034, Joint FAO/WHO Expert Committee on Food Additives (JECFA), 86th meeting 2018). Spirulina contains all eight amino acids essential to humans, has a digestibility range of 80–90%, and contains 60–70% protein on a dry weight basis, which is higher than any other natural food [15,16,17,18,19]. Within its 6–9% fat content, spirulina is rich in unsaturated fatty acids such as linoleic acid, docosahexaenoic acid, eicosapentaenoic acid, arachidonic acid, and stearidonic acid. Moreover, spirulina contains moderate amounts of vitamin A, vitamin C, vitamin E, vitamin B12, thiamine, nicotinamide, pyridoxine, riboflavin, and folic acid. In addition, it has many functional bioactive ingredients, including phenolic phytochemicals, phycobiliprotein, and chlorophyll, that have antioxidant and anti-inflammatory properties [20,21]. It has a high content of total phenolic compounds such as catechin hydrate, epicatechin, pyrocatechol, C-phycocyanin and β-carotene, which contribute to the major antioxidant activity of spirulina. The phenolic compounds present in spirulina are primarily involved in the redox mechanism and function to prevent the formation of reactive oxygen species (ROS), eventually inhibiting inflammatory responses via anti-oxidative and anti-inflammatory mechanisms that have protective effects against various human diseases such as mild chronic inflammatory disease [22,23,24,25]. 

Previous studies have shown that spirulina can be a good protein source for populations in developing countries that are vulnerable to protein malnutrition as it can support body growth, avoid fatty liver development associated with protein deficiency, and improve the nutritional status of malnourished humans [26,27,28,29,30,31,32,33,34]. Spirulina has also been shown to prevent fatty infiltration of the liver in diabetic rats by inhibiting adipogenesis and lipogenesis [35]. In addition, it has been shown to lower blood cholesterol levels associated with a high-cholesterol diet in animal models and to prevent arteriosclerosis [36,37]. However, similar or greater effects on bone growth and bone strength under adequate nutritional conditions, such as an alternative vegetable protein source compared to an animal protein source, have not been reported. 

The height of children is rapidly becoming a social issue as there is an increase in the number of people who are concerned about their child’s height. As a result, GH therapy is being indiscriminately used without adequate protection against side effects [38,39,40,41,42,43,44,45,46,47]. This study investigated the beneficial effect of using spirulina as a dietary supplement on skeletal growth and growth-related hormone levels in growing male rats. The results indicated that a spirulina supplement can enhance bone growth and bone strength and provide an antioxidant protective effect against tissue damage. 

## 2. Materials and Methods 

### 2.1. Animal Care and Diets

Three-week-old male Sprague–Dawley rats weighing approximately 50 g were used in this study (Damulscience, Daejeon, Korea). The animals were divided into four groups by applying a randomized design, and each group contained 17 rats (Table 1). All rats were housed in a room with constant temperature (23 ± 1 °C) and relative humidity (50 ± 5%) conditions and under a 12-hour light/dark cycle. 

Freeze-dried spirulina (*S. platensis*) powder obtained from Dainippon Ink and Chemicals, Inc. (Tokyo, Japan) was kindly donated by ES Biotech Co. (Cheonan, Korea) for use in this study. The composition of the spirulina powder is summarized in Table 2. The control group was fed with a normal AIN93G rodent diet while the other groups were fed an AIN93G diet with a portion of the diet replaced by spirulina powder. The protein source in the control group diet was 100% casein. Portions of the casein protein within the AIN93G diet were replaced with spirulina protein; three treatment groups, SP30, SP50, and SP70, were fed AIN93G diets with 30%, 50%, and 70%, respectively, of the casein replaced by spirulina. To ensure that total calorie (kcal) content and total amounts of vitamins, minerals, fiber, carbohydrate, and protein (g) per 100 g of each diet formula were similar among the groups, adjustments were made to the control diet (Table 3). The same amount of food was given to each group and the animals were weighed every 2 days. The food efficiency ratio (FER) was determined by measuring the total increment of animal weight and total diet intake and calculating FER as FER = total weight increment (g)/total diet intake (g).

All animal experiments were approved by the Committee of Animal Care and Experiment of Chungnam National University (Daejeon, Korea) with reference number (CNU-00036) and were carried out in accordance with the National Institutes of Health Guide for the Care and Use of Laboratory Animals (NIH Publications No. 8023, revised 1978).

### 2.2. Tissue Collection and Preparation 

Five animals from each group were fasted overnight before sacrifice at 0, 3, and 7 weeks. Blood samples were taken from the carotid artery and kept in a heparin-treated test tube. Plasma was collected by centrifugation for 15 minutes at 1000 *g* and then stored at –70 °C until analysis. Liver, heart, kidney, and adipose tissues were extracted, rinsed with 0.9% PBS, and weighed. The femur and lumbar spine were isolated and muscles, fat, and ligaments were removed. The length of the femur and lumbar spine were measured using a Digimatic caliper (Mitutoyo, Japan). Weight, bone strength, and mineral content of the femur and lumbar spine were determined.

### 2.3. Bone Strength and Bone Mineral Content (BMC) Measurement 

The breaking force of the femur and lumbar spine was measured by using a texture analyzer (TA/XT2, Stable Micro System, England). For bone measurement, the plunger of the texture analyzer was arranged appropriately and its placement was adjusted to the middle of the femur. The plunger was then dropped in order to fracture the bone. The plunger drop conditions used for breaking the femur and lumbar spine were: distance time 80%, pre-test speed 2.0 mm/s, test speed 1.0 mm/s, and post-test speed 5.0 mm/s. 

For analysis of mineral content of the bones, 0.5–1 g of femur and lumbar spine were placed in a container and 7 mL 65% HNO_3_ and 1 mL 30% H_2_O_2_ were added. The calcium and magnesium were diluted by LaCl_3_ and the potassium was diluted by distilled water. The mineral content in the diluted solutions was measured by an atomic absorption spectrophotometer (ICP Microphone). 

### 2.4. Biochemical Analysis

Several serum factors were measured by using analysis kits as follows: GH (Rat Growth Hormone ELISA; acceptable range 50% blank/maximum binding: 3.6 ng/mL, Cayman, MI, USA); IGFBP-3 (Rat IGFBP-3 ELISA; sensitivity: 0.09 ng/mL, BioVendor, Brno, Czech); IGF-1 (Rat IGF-1 ELISA; assay range: 10~1200 ng/mL, IDS Ltd. Bolden, UK); Osteocalcin-3 (ELISA; sensitivity: 1 ng/mL, BTI, NM, USA); Parathyroid hormone (Intact-PTH Rat EIA; sensitivity: 1.57 pg/mL, DRG. Inc., NJ, USA); total cholesterol, HDL, and LDL kit (Enzyme kit; accuracy, r = 0.987, YD Diagnostics, Yongin, Korea); alanine aminotransferase (ALT) kit (AM 101-K Kit, Asan Pharm. Co. Ltd., Hwaseong, Korea). Calcium, phosphorus, lactate dehydrogenase (LDH), and glucose concentration in plasma were measured by using a blood chemistry analyzer (ARCO-PC, Biotecnica Ins., Italy). The concentration of deoxypyridinoline (DPD) in urine was analyzed by using a deoxypyridinoline assay kit, and creatinine was analyzed by using a creatinine assay kit (QUIDEL; creatinine accuracy: r = 0.993; DPD sensitivity: 1.1 nM/L, CA, USA).

### 2.5. Antioxidant Enzyme Activities and Lipid Peroxide Measurement

Glutathione (GSH), 5,5’-dithio-bis-nitrobenzoic acid (DTNB), Trizma (Tris base), glutathione reductase (GR), and oxidized glutathione (GSSG) were purchased from Sigma-Aldrich Chemical (Sigma-Aldrich Chemical Co., St. Louis, USA). The antioxidant enzyme activities were calculated to 1 g protein content by applying the method of Bradford and using BSA as a standard. The quinone reductase (QR), Glutathione *S*-transferase (GST), GR, and GSH concentrations of liver tissue were measured by applying the following methods. The QR activity in the tissues was measured with 25 mM Tris-HCl buffer supplemented with BSA, FAD, 0.1 mM NADPH, and 10% PMS. The mixture was measured at 600 nm with a spectrophotometer. The GST activity was determined by mixing the tissue homogenate with 1-chloro-2,4-dinitrobezene and measuring activity at 340 nm with a spectrophotometer. The tissue homogenate for GR activity was reacted with 26.98 mM EDTA in 0.1 M Tris-HCl buffer supplemented with 66.0 mM GSSG and 9.18 mM NADPH and measured by determining the absorbance by spectrophotometer at 340 nm. The GSH activity was determined by mixing the tissue homogenate with 0.1 M potassium phosphate buffer with 10 mM DTNB, and 5 mM NADPH, equilibrated for 1 min by adding one unit of GR and measuring the absorbance at 412 nm with a spectrophotometer. A 0.04 mM GSH was used to obtain a standard curve. 

For lipid peroxide measurement, the blood and organs (liver, kidney, heart) were placed on ice, and homogenized with 50 mM sodium phosphate buffer using a tissue homogenizer with a Teflon pestle (Dupont, Wilmington, DE, USA). One mL of homogenate was mixed with 1 mL 8.1% SDS, 2 mL 20% acetic acid, and 1 mL 0.75% TBA and the mixture then boiled for 30 min. The absorbance of the malondialdehyde (MDA)-TBA adduct formed in the supernatant was measured colorimetrically at 532 nm. The value of MDA was calculated from a standard curve prepared using tetramethoxypropane (TMP) and is expressed as a thiobar–bituric acid reactive substance (TBARS) value.

### 2.6. Statistics 

All results were expressed as mean ± SEM and analyzed one-way ANOVA or *t*-test using SPSS 24.0 (Statistical Package for Social Science, SPSS Inc., Chicago, IL, USA) software package program and GraphPad Prism 8 (GraphPad Software Inc., CA, USA) software. T-test was used to compare a treatment group with the control group. Biochemical changes between the groups within the same week or between weeks within the same group were investigated using ANOVA analysis, with which a post-hoc comparison was made with Least Significant Difference (LSD) and Duncan’s multiple-range test. Statistical significance refers to results where * *p* < 0.05, ** *p* < 0.01, or *** *p* < 0.001 for the comparison between the weeks within a group and ^#^ *p* < 0.05, ^##^ *p* < 0.01, or ^###^ *p* < 0.001 for the comparison between the groups in the same week were obtained.

## 3. Results

### 3.1. Body Weight, Food Intake, Tissue Damage, and Blood Lipid Profile 

**Figure 1:**
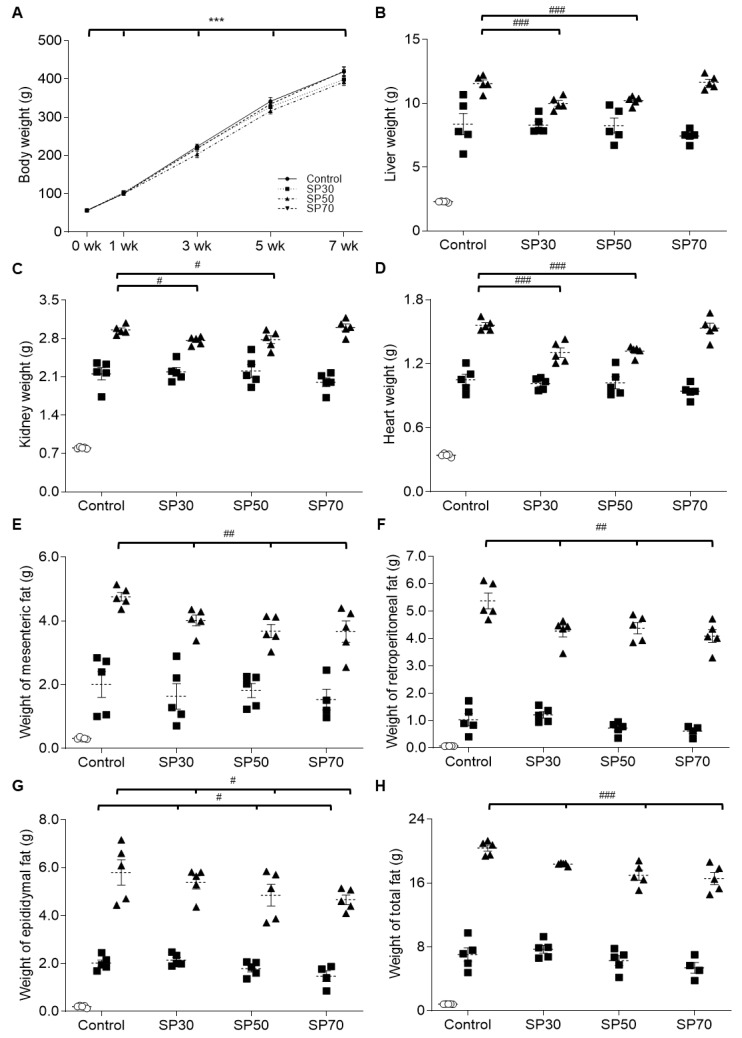
Effect of spirulina on the weight of body, organs, and fat tissues. Weights of (**A**) body, (**B**) liver (g/100 g BW), (**C**) kidney (g/100 g BW), (**D**) heart (g/100 g BW), (**E**) mesenteric fat (g/100 g BW), (**F**) retroperitoneal fat (g/100 g BW), (**G**) epididymal fat (g/100 g BW), and (**H**) total fat (g/100 g BW) were measured in growing male rats over the 7-week course of the study. Data are mean ± SEM values. Significant differences were determined using *t*-tests or one-way ANOVA with post hoc Duncan’s multiple-range test comparisons. *** *p* < 0.001 between weeks within a group and ^#^ *p* < 0.05, ^##^ *p* < 0.01, or ^###^ *p*< 0.001 between groups in the same week. Control: AIN 93G diet; SP30: 30% of protein source replaced with spirulina; SP50: 50% of protein source replaced with spirulina; SP70: 70% of protein source replaced with spirulina. The week of the treatment is represented by O: 0 weeks; ■: 3 weeks; ▲: 7 weeks in each group.

The body weight, food intake, and dietary efficiency of rats fed the experimental diets for 7 weeks were measured. Figure 1A shows that average body weight increased significantly over 7 weeks in each group. However, there were no significant differences between the groups at 0, 3, and 7 weeks (Table 4). In addition, there was no significant difference in the amount of food intake or the FER among the groups after 3 and 7 weeks (Table 4). 

Since spirulina was used as a substitute protein source, levels of urinary creatinine and DPD as kidney damage markers were examined. As shown in Figures S1A,B, both markers showed significant dose-dependent decreases with the SP70 group having the lowest levels of both markers, suggesting that the protein excretion effect is relieved by a spirulina-supplemented diet. Blood albumin (Figure S1C) and blood glucose level (Figure S1D) showed no significant difference between the spirulina substitution diets and the control diet at 7 weeks, suggesting that there were no influences on protein homeostasis in the circulatory system or on insulin resistance. 

Alanine aminotransferase (ALT) assessment for liver damage showed no significant difference between the spirulina substitution diets and the control diet at 7 weeks (Figure S1E). Elevated lactate dehydrogenase (LDH) can indicate tissue damage by disease or stress related to organ growth. In this study, plasma LDH levels increased over time in all groups as expected, but compared to the other groups, the SP70 group showed a significant LDH reduction after 7 weeks of treatment, suggesting that spirulina supplementation might protect the body against tissue stress due to growth (Figure S1F).

Blood lipid analyses, including total cholesterol, HDL, and LDL, revealed no significant differences between the spirulina substitution diet and the control diet (Figure S2). 

In conclusion, the results suggest that spirulina has potential as a candidate protein substitute as there were no indications of toxic effects related to kidney damage, liver damage, blood glucose changes, or changes in lipid profiles. 

### 3.2. Change of Organ Growth 

The effect of spirulina on organ weight of the growing rats over the 7-week treatment period was investigated. Liver (Figure 1B), kidney (Figure 1C), and heart (Figure 1D) weights increased significantly over time in each group, as expected. At 7 weeks, there were no differences in weight gains in the liver, kidney, and heart tissues between the control group and the SP70 group; however, the SP30 and SP50 groups showed significantly less growth than that of the control group, suggesting that 70% of protein source replaced with spirulina might be the most effective spirulina supplementation level. 

### 3.3. Fat Accumulation during the Growth

The effect of spirulina on the weight change of adipose tissues in the growing rats was also examined. Adipose tissues in all groups showed significant increases during the experimental period (*p* < 0.001). Interestingly, the weight gain of mesenteric fat (Figure 1E), retroperitoneal fat (Figure 1F), epididymal fat (Figure 1G), and total fat (Figure 1H) decreased significantly in a spirulina dose-dependent manner. In particular, the weight increase of epididymal fat was notably low in the third measurement week. At 7 weeks, SP70 showed the lowest weight gains in all fat tissues, and the gains were significantly lower than those in the other groups. These observations suggest that a high level of spirulina substitution can prevent the accumulation of fat without interfering with the weight gain of the rest of the body during growth. 

### 3.4. Improvement of Length and Weight of Femur and Lumbar Spine by Spirulina 

**Figure 2:**
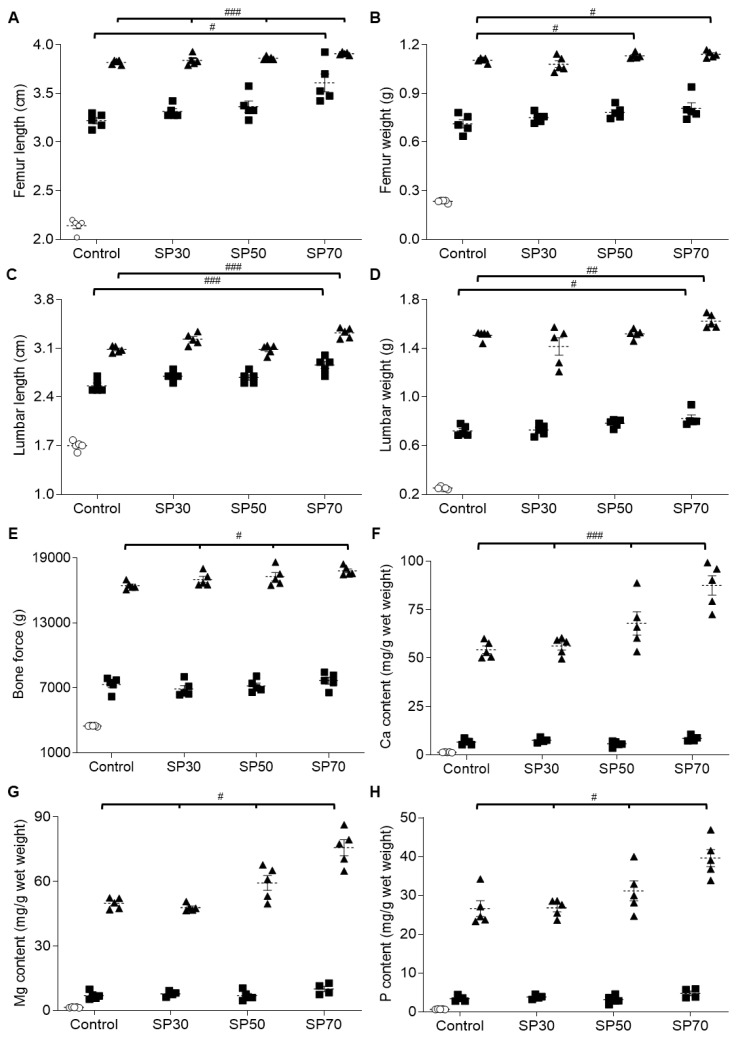
Effect of spirulina on bone growth, bone strength, and bone mineral content (BMC). (**A**) Femur length, (**B**) femur weight, (**C**) lumbar spine length, (**D**) lumbar spine weight, (**E**) breaking force of femur, (**F**) Ca content of femur, (**G**) Mg content of femur, and (**H**) phosphate content of femur were measured in growing male rats over the 7-week course of the study. Data are mean ± SEM values. Significant differences were determined using *t*-tests or one-way ANOVA with post hoc Duncan’s multiple-range test comparisons. ^#^ *p* < 0.05, ^##^ *p* < 0.01, or ^###^ *p* < 0.001 between groups in the same week. Control: AIN 93G diet; SP30: 30% of protein source replaced with spirulina; SP50: 50% of protein source replaced with spirulina; SP70: 70% of protein source replaced with spirulina. The week of the treatment is represented by O: 0 weeks; ■: 3 weeks; ▲: 7 weeks in each group.

Next, the lengths and weights of the femur and lumbar spine were measured. As expected, the lengths and weights of the femur and lumbar spine increased significantly over the 7-week growth period in each group. At 7 weeks, the femur lengths (Figure 2A) and weights (Figure 2B) of the SP50 and SP70 groups increased significantly compared to those of the control group. However, even at 3 weeks, the femurs of the SP70 group were significantly longer than those of the control group. Also, the lumbar length (Figure 2C) and weight (Figure 2D) of the SP70 group were significantly greater than those of the control group after both 3 weeks and 7 weeks of treatment. These results indicate that the high spirulina content in the SP70 group significantly increased the weight and length of both femur and lumbar spine bones consistently over a 7-week period, resulting in enhanced bone development in growing male rats. 

### 3.5. Enhanced Bone Strength via Higher Bone Mineral Content by Spirulina

The femur bending strengths of the rats are shown in Figure 2E. Although bone strength was increased significantly over time in all groups, the SP-substituted groups showed significantly greater bone strength at 7 weeks. Moreover, the increases were SP dose-dependent. The bone strength of the SP70 group was significantly higher (5.3% higher) than that of the control group at 7 weeks (*p* < 0.05). The results indicate the enhancing effect of spirulina on bone strength. 

Next, the mineral content of the femur bones was measured. The Ca (Figure 2F), Mg (Figure 2G), and P (Figure 2H) content in the femurs significantly increased during the experiment period in all groups, as expected. However, at 7 weeks, the mineral contents of all three SP substitution groups were significantly higher than that of the control group, and the BMCs changed in a dose-dependent manner, suggesting a positive effect of spirulina on bone strength. 

### 3.6. Increased plasma Growth Hormone, IGF-1, and IGFBP-3 Levels by Spirulina

**Figure 3:**
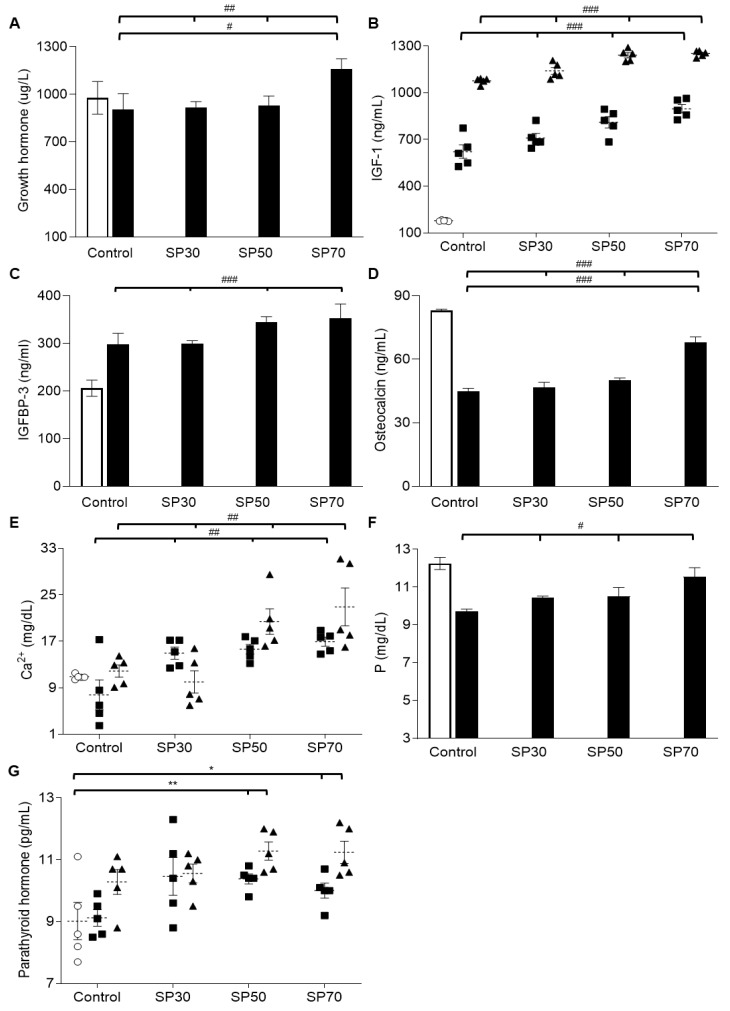
Effect of spirulina on growth hormones and related hormones in blood. Plasma (**A**) Growth hormone (GH), (**B**) insulin-like growth factor 1 (IGF-1), (**C**) insulin-like growth factor binding protein 3 (IGFBP-3), (**D**) osteocalcin, (**E**) free calcium, (**F**) free phosphate, and (**G**) parathyroid hormones (PTHs) were measured in growing male rats over the 7-week course of study. Data are mean ± SEM values. Significant differences were determined using *t*-tests or one-way ANOVA with post hoc Duncan’s multiple-range test comparisons. * *p* < 0.05, ** *p* < 0.01 between weeks within a group and ^#^ *p* < 0.05, ^##^ *p* < 0.01, or ^###^ *p* < 0.001 between groups in the same week. Control: AIN 93G diet; SP30: 30% of protein source replaced with spirulina; SP50: 50% of protein source replaced with spirulina; SP70: 70% of protein source replaced with spirulina. The week of the treatment is represented by O: 0 weeks; ■: 3 weeks; ▲: 7 weeks in each group. White bar: 0 weeks; black bar: 7 weeks.

Since we observed enhancement of bone growth and bone strength by spirulina treatment, we investigated whether the growth-regulating hormonea level was influenced by spirulina (Figure 3). Although the GH level was steady throughout the experiment in the control group, the spirulina-fed groups showed continuous increases in GH level over the 7-week study in a dose-dependent manner (Figure 3A). This result suggests that spirulina treatment can increase GH levels over time, potentially allowing bones to grow continuously. 

IGF-1 is an anabolic hormone with a structure similar to that of insulin and regulates the linear and microarchitectural growth of bones, in particular in osteoblasts. Therefore, we examined the plasma level of IGF-1 (Figure 3B). Plasma IGF-1 levels increased significantly during the experiment period in each group, as expected. However, plasma IGF-1 levels in the spirulina-fed groups were significantly higher than those of the control group after both 3 weeks (*p* < 0.001) and 7 weeks (*p* < 0.001) of treatment. 

Insulin-like growth factor binding protein 3 (IGFBP-3) is the main IGF transport protein in the blood and most dependent on IGF-1. Therefore, in this study, we examined the IGFBP-3 level in plasma. As shown in Figure 3C, IGFBP-3 levels in each of the SP groups were significantly higher than those of the control group at 7 weeks, mirroring the IGF-1 results. 

These results suggest that spirulina, especially in high amounts (SP70), enhances the release of GH, followed by a continuing increase in the release of IGF-1, ultimately resulting in the SP-related bone growth enhancements observed above.

### 3.7. Increased Plasma Osteocalcin, Mineral, and Parathyroid Hormone (PTH) Levels Dose-Dependently

Osteocalcin is a noncollagenous calcium-binding protein hormone secreted by osteoblasts. High plasma osteocalcin levels correlate relatively well with increases in bone mineral density during bone formation, which can be used as a preliminary biomarker of bone formation. Therefore, we examined the effect of spirulina on plasma osteocalcin (Figure 3D). While the osteocalcin level decreased significantly in the control group over the experimental period, spirulina treatment significantly increased the osteocalcin level over time compared to the control group (*p* < 0.001); moreover, the increases were dose-dependent. These data suggest that spirulina increases the levels of osteocalcin in plasma, resulting in enhanced bone mineral density and bone strength in growing rats, as observed above.

Next, we examined the effect of spirulina on blood mineral concentrations in growing rats. The spirulina-fed groups had significantly higher levels of free calcium concentrations (*p* < 0.01) compared to the control group (Figure 3E). The increases were dose-dependent.The serum phosphorus (P) level decreased over 7 weeks in the control group, but the spirulina-fed groups showed continuous increases in P level dose-dependently (Figure 3F). 

Parathyroid hormones (PTHs) regulate the plasma calcium level through its effects on bone, kidney, and intestine [48]. Therefore, we examined PTH levels to determine whether spirulina affects calcium level via PTH activities. Interestingly, plasma PTH level only increased significantly in the SP50 and SP70 groups over the course of the study; there was no significant increase in PTH level in either the control or SP30 groups (Figure 3G). Overall, these data suggest that higher doses of spirulina can increase the PTH level, which is associated with an increase in the free calcium available for bone mineralization in plasma. 

### 3.8. Increased Antioxidant Enzyme Activities and Reduced Lipid Peroxidation by Spirulina 

**Figure 4:**
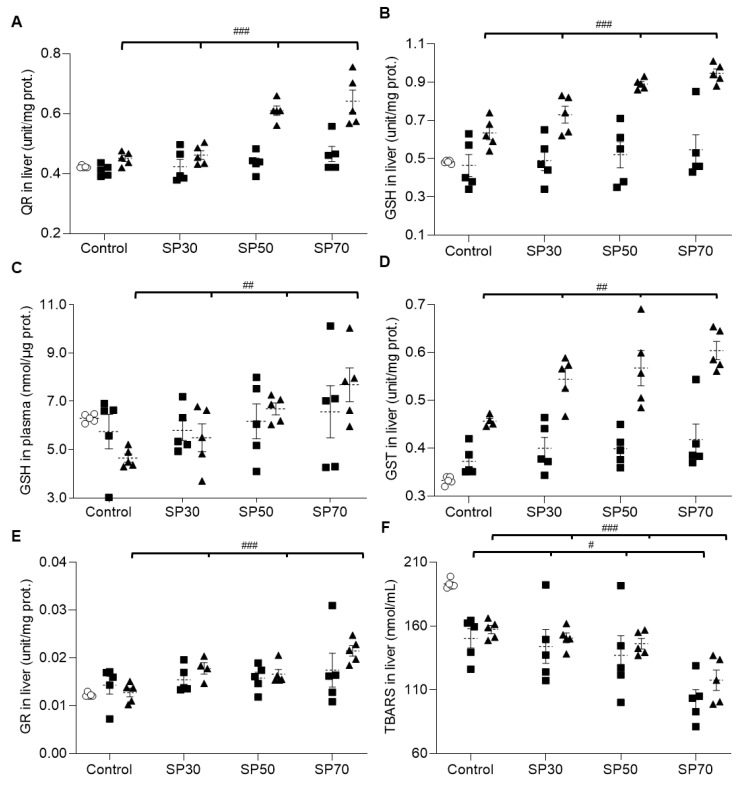

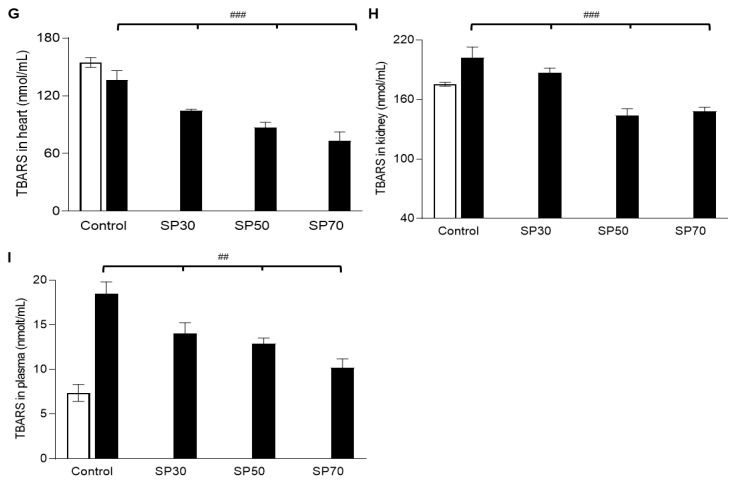
Effect of spirulina on antioxidant activities and lipid peroxidation. (**A**) quinone reductase (QR) in liver, (**B**) glutathione (GSH) in liver, (**C**) GSH in plasma, (**D**) glutathione *S*-transferase (GST) in liver, (**E**) glutathione reductase (GR) in liver, (**F**) thiobar–bituric acid reactive substance (TBARS) in liver, (**G**) TBARS in heart, (**H**) TBARS in kidney, and (**I**) TBARS in plasma were measured in growing male rats fed with control, SP30, SP50, and SP70 diet over the 7-week course of the study. Data are mean ± SEM values. Significant differences were determined using one-way ANOVA with post hoc Duncan’s multiple-range test comparisons. ^#^ *p* < 0.05, ^##^ *p* < 0.01, or ^###^ *p* < 0.001 between groups in the same week. Control: AIN 93G diet; SP30: 30% of protein source replaced with spirulina; SP50: 50% of protein source replaced with spirulina; SP70: 70% of protein source replaced with spirulina. The week of the treatment is represented by O: 0 weeks; ■: 3 weeks; ▲: 7 weeks in each group. White bar: 0 weeks; black bar: 7 weeks.

Figure 4 shows the effect of spirulina on antioxidant enzyme activities in growing rat tissues. While quinone reductase (QR) and glutathione (GSH) levels in the liver did not change significantly over time in the control group, the SP50 and SP70 groups showed significant increases in QR and GSH over time (Figure 4A,B). Then, we looked at the plasma GSH level to determine whether it reflected the level of GSH released from the liver. As shown in Figure 4C, there was no statistically significant change in plasma GSH level over the 7 weeks in each group. However, at 7 weeks there were significantly higher GSH levels in the spirulina-fed groups, especially in the SP70 group, than in the control group. 

Glutathione *S*-transferase (GST) (Figure 4D) and glutathione reductase (GR) (Figure 4E) levels in the liver also increased over time even in the control group, but at 7 weeks the GST levels in the spirulina-fed groups were significantly higher, in a dose-dependent manner, than that in the control group, thus indicating that spirulina enhances antioxidant activities in the liver and plasma.

TBARSs are formed as a by-product of lipid peroxidation. Therefore, lipid peroxidation was examined by measuring TBARS levels in various tissues of the growing rats. TBARS in the liver decreased significantly over the experimental periods (Figure 4F). However, at both 3 and 7 weeks, TBARS levels in the spirulina-fed groups decreased significantly in a dose-dependent manner from that of the control group. The TBARS levels in heart, kidney, and plasma showed the same patterns as those of TBARS levels in the liver (Figure 4G–I) at 7 weeks. 

Overall, these data suggest that a higher content of spirulina is more effective in enhancing antioxidant activities and inhibiting lipid peroxidation in various tissues. 

## 4. Discussion

Spirulina has been well considered an excellent protein source for malnourished children since it contains all eight essential amino acids and is composed of 60–70% protein (based on dry weight). However, no studies so far have reported on the effect of spirulina as a protein substitute on growth under normal dietary conditions. Therefore, we hypothesized that spirulina, a plant-origin protein, could be a functional substitution for animal protein, in our case, casein protein. 

Our results show, for the first time, that spirulina was able to enhance bone growth, bone strength, BMC, and antioxidant activities by regulating GH, IGF-1, osteocalcin, and PTH in normal nutritional conditions; moreover, the higher the spirulina content, the greater the positive effect. These results indicate that, compared to the control and low concentration of spirulina substitution, a 50% or 70% spirulina protein substitution may provide more favorable effects on osteogenesis. 

In addition, the blood lipid profile, liver function, and kidney function were not affected by the spirulina diet. Interestingly, lactate dehydrogenase (LDH) levels in the blood, a marker of tissue damage, were significantly lower in the SP70 group than in the other SP and control groups, suggesting that a high dietary content of spirulina might protect the body from tissue damage. 

The hormonal changes observed in our model confirmed that IGF-1 is a key endocrine factor involved in bone growth modulation that can be altered by nutritional challenge. However, these hormonal alterations did not fully explain how spirulina supplementation could enhance the biomechanical properties of bones and should be studied further. 

Surprisingly, there was less accumulation of epididymal, retroperitoneal, and mesenteric fat in rats fed a spirulina-supplemented diet than in those fed the control diet, suggesting that spirulina could inhibit fat accumulation without interfering with normal body and organ growth. 

It was shown that a low protein diet decreases the expression of the Sirt1 gene, followed by reduced PPARα signaling, resulting in fatty liver [49]. Also, a recent study demonstrated that liver-specific disruption in GH signaling leads to a fatty liver [50]. Another study showed that spirulina could prevent the negative effects associated with reduced circulating IGF-1 levels and high hepatic fat content in a protein deficiency model [51] as well as animal models of diabetes and non-alcoholic steatohepatitis [32]. 

Another player in the hormonal regulation caused by spirulina would be osteocalcin. Osteocalcin, also stimulates pancreatic -cells to release more insulin, increasing insulin sensitivity [52,53,54,55,56]. Our data show that while osteocalcin levels decreased significantly in the control group over the experimental period, spirulina treatment significantly increased osteocalcin levels over time (*p* < 0.001); moreover, the increases were dose-dependent. Also, blood glucose levels in our study decreased dose-dependently by spirulina, but not significantly. Therefore, our data might help explain the mechanism through which spirulina prevents the development of hepatic GH resistance, followed by the reduction of fatty infiltration into the liver via regulating osteocalcin and growth hormones.

We also examined whether spirulina can protect the growing body from tissue damage caused by oxidation. The antioxidant enzyme activities of the liver, the Glutathione (GSH), glutathione reductase (GR), Glutathione *S*-transferase (GST), and quinone reductase (QR) activities were observed to be positively dependent on the amount of spirulina supplementation. Although plasma GSH did not show a marked increase with spirulina treatment, GSH levels in the liver were significantly increased by spirulina feeding in a dose-dependent manner. Moreover, lipid peroxidation in the liver, plasma, kidney, and heart tissues was significantly lower in spirulina-fed groups than in the control group at 7 weeks of treatment. 

It was reported that spirulina exhibits antioxidant properties due to containing various phenolic compounds [57]. Although an analysis study revealed that the distributions of the total phenolic compounds varied between commercial products [23], it was claimed that chlorogenic acid, synaptic acid, salicylic acid, trans-cinnamic acid, and caffeic acid were commonly present in spirulina [58]. The antioxidant compounds in spirulina, such as phycobilins and phycocyanins, also inhibit the activities of catalytic enzymes, such as lipoxygenase and cyclooxygenase, or enhance the activity of enzymes, such as glutathione peroxidase, catalase, and superoxide dismutase [59,60]. These polyphenols were reported to have anti-inflammatory, antiviral, antioxidant, antithrombotic, vasodilatory, antidiabetic, neuroprotective, hepatoprotective, and anticarcinogenic properties [21,22,24,28,29,32,35,36,37,59,60,61,62,63]. However, the metabolic pathways for the formation of phenolic compounds in spirulina and their importance are still unknown [64]. 

Another beneficial component of spirulina is γ-linolenic acid. It was discovered that the amounts of γ-linolenic acid ranged from 0.16 g/100 g to 1.24 g/100 g and accounted for an average of 14% of the total polyunsaturated fatty acids in spirulina [65]. Many in vitro studies confirmed that **γ**-linolenic acid can be used to effectively lower cholesterol and treat atopic eczema, breast cancer, and premenstrual disorder [66,67,68]. 

In humans, longitudinal growth occurs until the epiphyseal plate becomes ossified in the late teens and early twenties [69]. However, rat skeleton is considered fully mature only after the age of 10 months. At the age of 10 months, peak bone mass is achieved, and the total longitudinal bone growth stops. Bone growth in the proximal tibia and distal tibia epiphysis stops at the age of 15 and 3 months, respectively, whereas lumbar vertebrae continues to grow for up to 21 months [14]. However, similar to the human skeleton, the rat skeleton shows a gradual transition from modeling to remodeling that is related to age progression and cessation of longitudinal bone growth in both cancellous and cortical bone [70,71,72,73,74]. 

The Food and Drug Administration requires that novel therapies in bone research must be tested both in rodents (preferably rats) and in a large animal model [75,76]. The reason for using a second species in preclinical skeletal research is the lack of the Haversian system in rodents. A potential drawback of our study to be implicated into the human is the lack of Haversian remodeling in the rat skeleton. Therefore, this difference might affect our finding when applied to the clinical setting.

For the first time, our results showed that growing rats fed a 70%/30% spirulina/casein protein diet instead of a 100% casein protein diet enhanced bone development, antioxidant activities, and minimal fat accumulation in adipose tissues without toxicity, suggesting that substitution of an animal protein source with a plant protein source such as spirulina can be beneficial. It implies that long-term dietary supplementation with spirulina from infancy to early childhood might help promote growth and lengthen the growth period. 

Recently, it was found that human gut bacteria can synthesize proteinogenic amino acids and produce a range of metabolites via protein fermentation, some known to exert beneficial or harmful physiological effects on the host [77,78,79,80,81,82,83]. It is still in an early stage whether the type and amount of dietary protein consumed affect the diversity and composition of the intestinal microbiota, and the luminal environment of the intestinal epithelium and peripheral tissues on the host health. However, major progress is expected in the near future. Therefore, it would be interesting to know how spirulina supplementation affects gut microbiome profiles and what beneficial metabolites from spirulina protein could be produced, and whether, ultimately, these improve gut health as well as systemic immunity.

## Figures and Tables

**Table 1:** Experimental design of the study.

Experimental Group	Diet	Number of Mice
Control	AIN93G	17
SP30	30% of AIN93G replaced by spirulina	17
SP50	50% of AIN93G replaced by spirulina	17
SP70	70% of AIN93G replaced by spirulina	17
Total		68

**Table 2:** Composition of freeze-dried powder of spirulina.

Composition			
Macronutrients (g/100 g)		Phytonutrients (mg/100 g)	
Calories (kcal/100 g)	360.70	Phycocyanin	8000.00
Moisture (%/100 g)	8.70	Chlorophyll a	1300.00
Carbohydrate	17.50		
Fat	4.30		
Protein	63.00		
Dietary fiber	6.50		
Vitamins (mg/100 g)		Minerals (mg/100 g)	
Vitamin A	2.95	Calcium	98.80
β-Carotene	177.00	Iron	40.90
Vitamin B1	3.08	Phosphorus	859.00
Vitamin B2	3.74	Magnesium	319.00
Vitamin B6	0.83	Zinc	1.28
Vitamin B12	0.18	Copper	0.32
Vitamin E	12.70	Manganese	3.77
α-Tocopherol	12.50	Chromium	0.06
β-Tocopherol	0.50	Potassium	1560.00
Vitamin K1	1.59		
Vitamin K2	0.08		
Folic acid	0.08		
Niacin	23.50		

**Table 3:** Composition of experimental diets.

Components (g/kg Diet)	Group			
	Control	SP30	SP50	SP70
Casein	200.00	140.00	100.00	60.00
Corn starch	397.49	377.28	363.81	350.34
Dyetrose	132.00	132.00	132.00	132.00
Sucrose	100.00	100.00	100.00	100.00
Cellulose	50.00	43.81	39.68	35.56
Soybean oil	70.00	65.91	63.18	60.45
t-Butylhydroquinone	0.01	0.01	0.01	0.01
Salt mix	35.00	31.06	28.43	25.80
Vitamin mix	10.00	9.20	8.66	8.13
L-cystine	3.00	3.00	3.00	3.00
Choline bitartrate	2.50	2.50	2.50	2.50
Spirulina	0.00	95.24	158.73	222.22
TOTAL	1000.00	1000.00	1000.00	1000.00
Total energy (kcal)	3948.07	3945.63	3944.00	3942.29
Nutrition from spirulina				
Energy (kcal)	0	355.24	592.06	828.81
Protein	0	60.00	100.00	139.99
Fat	0	4.10	6.83	9.56
Carbohydrate	0	16.67	27.78	38.89
Fiber	0	6.19	10.32	14.44
Total vitamin	0	0.80	1.34	1.87
Total mineral	0	3.95	6.57	9.20

**Table 4:** Body weight, food intake, and food efficiency ratio of the rats fed with spirulina.

		Control	SP30	SP50	SP70
3 weeks	Weight (g)	179.1 ± 11.0 ^NS^	188.7 ± 2.2	180.4 ± 5.3	176.9 ± 6.1
	Food intake (g/d)	24.6 ± 0.6 ^NS^	24.6 ± 0.4	24.4 ± 0.4	24.6 ± 0.4
	FER (%)	13.9 ± 0.6 ^NS^	13.0 ± 0.3	13.6 ± 0.2	13.9 ± 0.3
7 weeks	Weight (g)	378.9 ± 12.2 ^NS^	356.6 ± 11.8	341.8 ± 2.5	369.4 ± 13.4
	Food intake (g/d)	30.0 ± 1.0 ^NS^	28.3 ± 0.8	27.5 ± 0.6	26.7 ± 1.7
	FER (%)	8.0 ± 0.3 ^NS^	7.9 ± 0.2	8.1 ± 0.2	8.2 ± 0.3

All values are mean ± SEM. One-way ANOVA test at *p* < 0.05 by Duncan’s multiple-range test. NS: not significant. g/d: gram per day, FER (food efficiency ratio).

## Supplementary Materials

The following are available online at https://www.mdpi.com/2072-6643/12/4/1187/s1, Figure S1: Effect of spirulina on kidney function, liver function, or blood glucose level; Figure S2: Effect of spirulina on blood lipid profile.
