# Supplementary material for: Spirulina Enhances Bone Modeling in Growing Male Rats by Regulating Growth-Related Hormones
Source: Nutrients. 2020 Apr 24;12(4):1187. doi: 10.3390/nu12041187 (PMC7231069; doi:10.3390/nu12041187)
Supplement: Supplementary file 1 [file nutrients-12-01187-s001.pdf]

nutrients

MDPI

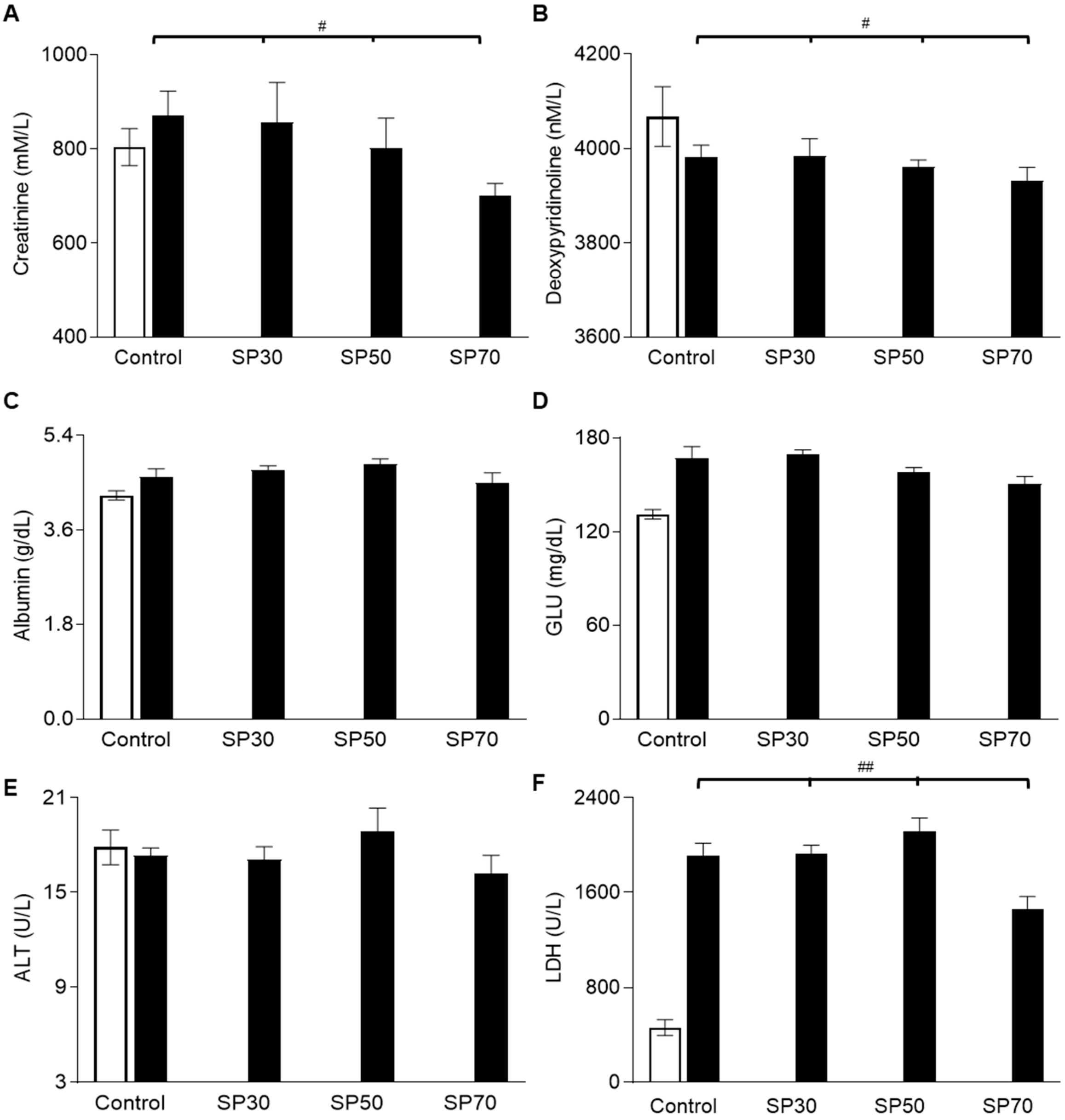

Figure S1. Effect of spirulina on kidney function, liver function, or blood glucose level. (A) Urinary creatinine, (B) urinary deoxypyridinoline, (C) plasma albumin, (D) blood glucose, (E) plasma ALT, and (F) plasma LDH level were measured at 0 and 7 weeks in growing male rats fed with control, SP30, SP50, or SP70 diets throughout the study. Data are mean ± SEM values. Significant differences were determined using one-way ANOVA with post hoc Duncan's multiple-range test comparisons. $^{\#}$ $p < 0.05$, or $^{\#\#}$ $p < 0.01$ between groups in the same week. Control: AIN 93G diet; SP30: 30% of protein source replaced with spirulina; SP50: 50% of protein source replaced with spirulina; SP70: 70% of protein source replaced with spirulina. White bar: 0 weeks, black bar: 7 weeks.

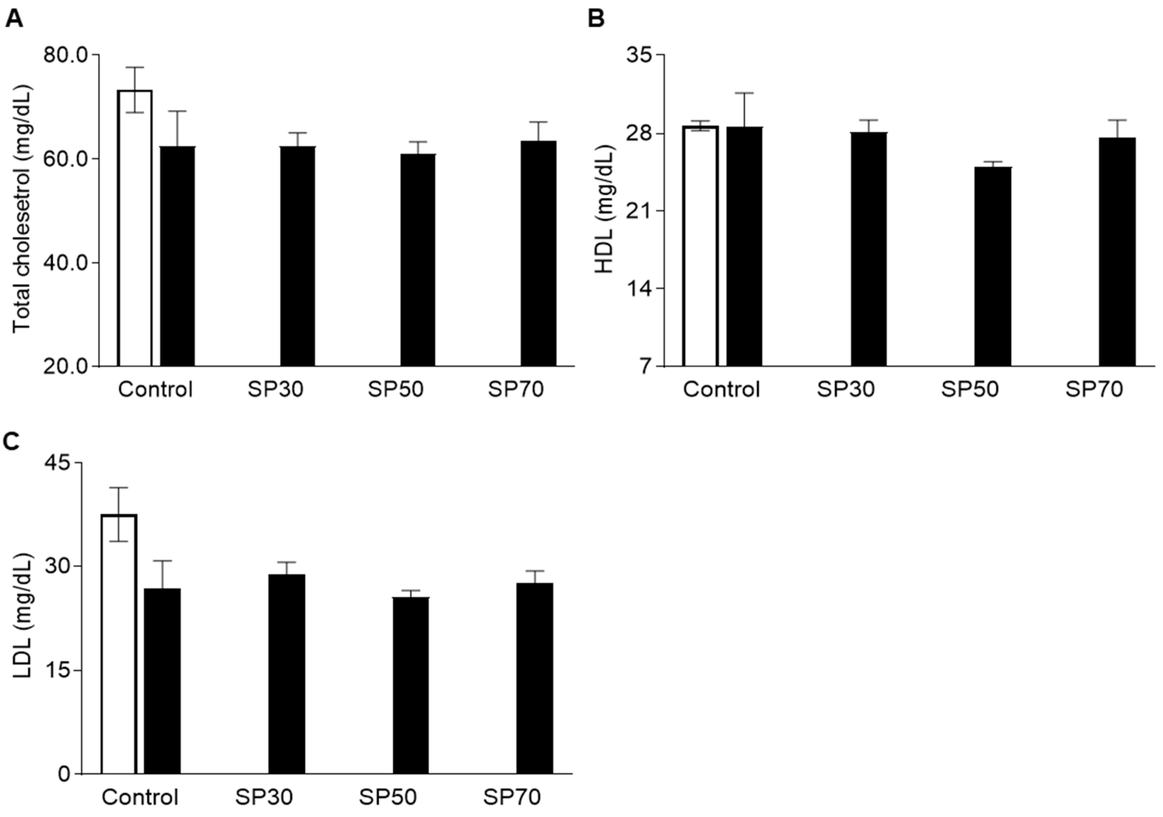

Figure S2. Effect of spirulina on blood lipid profile. (A) total cholesterol, (B) HDL, and (C) LDL levels were measured at 0 and 7 weeks in growing male rats fed with control, SP30, SP50, or SP70 diets throughout the study. Data are mean ± SEM values. Significant differences were determined using one-way ANOVA with post hoc Duncan's multiple-range test comparisons # $p < 0.05$ between groups in the same week. Control: AIN 93G diet; SP30: 30% of protein source replaced with spirulina; SP50: 50% of protein source replaced with spirulina; SP70: 70% of protein source replaced with spirulina. White bar: 0 weeks, black bar: 7 weeks.
